# Vacuum‐Assisted Closure Significantly Reduces Surgical Postoperative Complications Compared With Primary Abdominal Closure in Patients With Secondary Peritonitis: A Comparative Retrospective Study

**DOI:** 10.1002/wjs.12472

**Published:** 2025-01-10

**Authors:** Pooya Rajabaleyan, Ask Vang, Sören Möller, Sardar Khalaf, Anna Gosvig Ladegaard, Niels Qvist, Mark Bremholm Ellebæk

**Affiliations:** ^1^ Research Unit for Surgery Odense University Hospital Odense Denmark; ^2^ Open Patient Data Explorative Network (OPEN) Odense University Hospital and Department of Clinical Research University of Southern Denmark Odense Denmark

**Keywords:** open abdomen, secondary peritonitis, vacuum‐assisted closure

## Abstract

**Background:**

Vacuum‐assisted abdominal closure (VAC) is being increasingly used as an adjunctive procedure in the surgical treatment of secondary peritonitis. This study compared postoperative mortality and complication rates between VAC and primary abdominal closure (PAC).

**Method:**

This retrospective chart review included all patients diagnosed with secondary peritonitis who underwent laparotomy between 2010 and 2019. Data were collected from six hospitals within Southern Denmark, covering a population of approximately 1,225,000 inhabitants.

**Results:**

The study involved 315 patients (139 in the PAC and 176 in the VAC groups). In the VAC group, BMI, ASA, SOFA, MPI, and four quadrant contamination was significantly higher at the index operation. There were no significant differences in nonadjusted and adjusted postoperative mortality at 30 days, 90 days, and 1 year, with cumulative values of 13%, 16%, and 21%, respectively, compared with 16%, 21%, and 31%, in the PAC group (*p* = 0.519, *p* = 0.380, and *p* = 0.051, respectively). Cumulative adjusted surgical postoperative complications at 30 days, 90 days, and 1 year, as assessed by the comprehensive complication index, was significantly higher in the PAC group. Reoperations were significantly more common in the PAC group. The total length of the intensive care unit admission was significantly longer in the VAC group, with a mean of 9.0 ± 12.1 versus 6.7 ± 12.1 days (*p* < 0.001).

**Conclusion:**

VAC after laparotomy for secondary peritonitis did not significantly reduce mortality but increased ICU stay, whereas primary closure led to higher surgical complication rates and reoperations.

## Graphical abstract

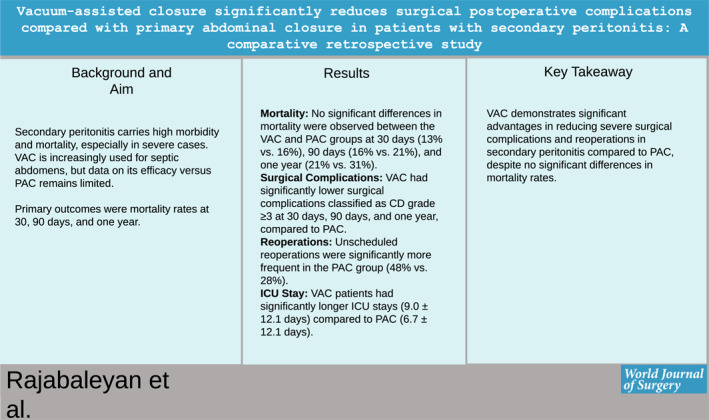

**Abbreviations**
APACHE II: Acute Physiology and Chronic Health Evaluation II
ASA: American Society of Anesthesiologists
CCI: comprehensive complication index
CD: Clavien–Dindo classification
CT: computed tomography
EAF: enteroatmospheric fistula
ICD‐10: International Classification of Diseases, Tenth Revision
ICU: intensive care unit
IQR: interquartile range
MPI: Mannheim peritonitis index
OPEN: Open Patient data Explorative Network
PAC: primary abdominal closure
PUD: percutaneous ultrasound‐guided drainage
REDcap: Research Electronic Data Capture
SOFA: Sequential Organ Failure Assessment
STROBE: Strengthening the Reporting of Observational Studies in Epidemiology
VAC: vacuum‐assisted closure
WHO: World Health Organization

## 1 Background

Secondary peritonitis results from direct contamination of the peritoneal cavity by spillage of gastrointestinal or urogenital contents [1, 2, 3]. Spillage of gastrointestinal contents, which accounts for more than 90% of cases, is the most common and serious source of contamination, leading to high morbidity and mortality rates that vary with the number of peritoneal quadrants involved [3]. A European study on fecal peritonitis reported a 28‐day mortality rate of 19.1%, which rose to 20.9% among patients in the intensive care unit (ICU), 28.7% by hospital discharge, and 31.6% at the 6‐month follow‐up [4, 5]. Fecal contamination and significant comorbidities are additional prognostic factors [6, 7, 8, 9, 10, 11]. Perforated appendicitis, the most common cause of peritonitis in younger patients and those with fewer comorbidities, carries a substantially lower risk of morbidity and mortality. Consequently, it is often excluded from studies on secondary peritonitis [12, 13].

The initial treatment strategies include patient resuscitation, empirical antibiotic therapy, source control surgery, and intensive care support [14, 15]. Laparotomy with definitive surgery followed by abdominal closure has been considered the gold standard for several decades [16]. However, the risk of severe postoperative complications remains high [17, 18, 19, 20] and diagnosing these complications may be difficult because of the risk of delayed reoperation, which often worsens the prognosis [6, 8, 11, 21]. The introduction of the open abdomen principle, well established in trauma surgery, has been associated with improved survival rates [22, 23, 24]. This principle is also applied in acute abdominal surgery for patients with other abdominal catastrophes, although the results are inconsistent [25, 26, 27, 28].

The main advantages of an open abdomen are early recognition and management of intra‐abdominal complications, effective drainage of the abdominal cavity, and a decreased risk of abdominal compartment syndrome [6, 7, 8]. Another potential benefit is a universally reduced inflammatory response [29, 30, 31]. Research in this field has described various techniques for covering the intestines [32]. The development of the vacuum‐assisted closure (VAC) system has revolutionized the management of open abdomen and is now widely used in acute abdominal surgery. Few cohort studies have compared primary closure with VAC treatment in the septic abdomen, primarily because of various limitations, and only a small number of case‒control studies involving relatively few patients have been performed [16, 33, 34, 35, 36, 37, 38, 39, 40, 41, 42, 43, 44, 45, 46, 47, 48, 49, 50, 51, 52, 53, 54, 55, 56, 57, 58, 59, 60, 61]. To our knowledge, no randomized controlled trial has been published comparing primary closure of the abdomen versus VAC, although two ongoing studies are currently recruiting patients [62, 63].

This study compared primary closure with on‐demand relaparotomy versus VAC with non‐mesh‐mediated fascial traction in patients with secondary peritonitis originating from the small bowel, colon, or rectum. The primary objective was mortality during hospitalization, at 30 days, at 90 days, and at 1 year. The secondary objectives were morbidity assessed using the comprehensive complication index (CCI) at 30 days, 90 days, and 1 year; the length of the hospital and ICU stay; the number of reoperations; the number of abscess drainages performed; and the number of abdominal computed tomography (CT) scans.

## 2 Materials and Methods

### 2.1 Study Design

We conducted a retrospective chart review of adult patients admitted in the region of Southern Denmark from 1 January 2010 to 31 December 2019, identified using International Classification of Diseases, Tenth Revision (ICD‐10) and procedure codes (Appendix 1). The study adheres to the Strengthening the Reporting of Observational Studies in Epidemiology (STROBE) guidelines for cohort studies [64].

### 2.2 Resource Use

Resource use was estimated by reviewing records from electronic patient journals including surgeries, hospital stay, ICU admissions, and radiological interventions. Unit costs were obtained from the Danish Health Authority, and diagnosis‐related groups were used to estimate hospital operating expenses.

### 2.3 Ethics

The study was approved by the Regional Data Protection Agency (ID: 21/21873) and the Regional Committees on Health Research Ethics for Southern Denmark (ID: 21/20419) in accordance with the Declaration of Helsinki.

### 2.4 Study Settings

The Southern Denmark region accommodates six medical centers providing acute surgical care for a population of approximately 1,225,000 residents.

### 2.5 Study Population

#### 2.5.1 Inclusion Criteria

Patients aged ≥ 18 years who underwent emergency laparotomy with preoperative findings of perforation originating from the small intestine, colon, or rectum were included in the study, provided either primary closure or VAC was applied at the index operation at the surgeon's discretion. The included conditions, classified by ICD‐10 codes, were Meckel's diverticulum; small bowel volvulus; anastomotic leakage; perforation of the small intestine, colon, or rectum; diverticulitis of the small or large intestine with perforation; volvulus; ileus; hernia with ileus; ischemic colitis; perforation following screening colonoscopy; and peritonitis.

#### 2.5.2 Exclusion Criteria

Patients with peritonitis originating from any other anatomical location, including acute peritonitis of other causes, were excluded from the study. Patients with abdominal trauma, acute mesenteric ischemia, chronic parenchymal liver disease, end‐stage metastatic disease, peritoneal carcinomatosis, peritoneal dialysis, pregnancy, a history of > 20 mg prednisolone daily, or any chemotherapy within 1 month prior to surgery were also excluded.

### 2.6 Operative Technique (VAC)

Non‐mesh‐mediated fascial traction with narrowing was employed for the VAC technique [62, 65]. A VAC Abdominal Dressing System (Vacuum‐assisted Closure System; Kinetic Concepts, Inc., San Antonio, TX, USA) was used for all patients. The visceral protective layer covered the intestines laterally, and foam was positioned above the dressing and extended subperitoneally up to 5 cm from the wound edge. An additional folded foam was placed within the laparostomy and covered with an adhesive drape. A 4 to 5 cm circular opening in the occlusive drape allowed the connection tubes to reach the vacuum pump (Vacuum‐assisted Closure System; Kinetic Concepts, Inc.). During vacuum application, the fascia was manually approximated by compressing it from both sides. Negative pressure of 125 mmHg was applied, and dressing changes were scheduled every 48 h until secondary closure could be performed.

### 2.7 Data Collection

The extracted data included preoperative baseline characteristics such as body mass index, age, American Society of Anesthesiologists (ASA) score, World Health Organization (WHO) performance status, smoking, alcohol consumption, Charlson comorbidity index, previous abdominal operations, time from symptom onset to surgery, and steroid use. Perioperative findings included the Mannheim peritonitis index (MPI), peritonitis etiology, perforation location, contamination extent (1–4 quadrants), and secondary fascial closure. The Acute Physiology and Chronic Health Evaluation II (APACHE II) score, which classifies disease severity based on patient age, chronic health status, and physiological parameters, and the Sequential Organ Failure Assessment (SOFA) score, which reflects organ dysfunction across six organ systems, were recorded during ICU stays. Postoperative complications were registered using the Clavien‒Dindo (CD) classification at 30 days, 90 days, and 1 year. Intra‐abdominal abscesses requiring percutaneous drainage, wound abscesses (detected by physical exam or CT), and hernias (identified via physical examination or CT within 1 year postoperatively) were also documented. Unplanned reoperations were recorded, whereas planned VAC changes were excluded from the surgical count to ensure that only emergent reinterventions were accurately captured. Data entry was performed in duplicate by two physicians, with discrepancies resolved by consensus, and the primary investigator (P.R.) verified the data through a double‐check process. All data were stored in Research Electronic Data Capture (REDcap) via Open Patient data Explorative Network (OPEN) at Odense University Hospital.

### 2.8 Statistical Analysis

Postoperative complications were reported as CCI scores and divided into surgical and medical complications using the CD classification. Numerical variables are presented as means with standard deviations or medians with interquartile ranges (IQRs), whereas categorical variables are shown as counts with proportions. The Wilcoxon rank‐sum test was used to compare numerical variables between groups, and linear regression with bootstrapped standard errors was applied to explore associations. Fisher's exact test was used for categorical data. Propensity score‐weighting analysis was conducted to adjust mortality (using Cox regression) and CCI outcomes for baseline characteristics (adjustments were made for age, time from diagnosis to operation, ASA classification, performance status, steroid use, Charlson comorbidity index, MPI, SOFA score, anatomical location of perforation, etiology, and whether treatment was provided at a secondary or tertiary center). Any patient in the PAC group, who later received a VAC treatment, remained in the PAC group for the statistical analysis, as the study was conducted as an intention to treat analysis.

A *p*‐value of < 0.05 was considered statistically significant. Statistical analysis was performed using STATA version 17.0 (StataCorp LP, College Station, TX, USA).

## 3 Results

### 3.1 Demographic Characteristics

**FIGURE 1:**
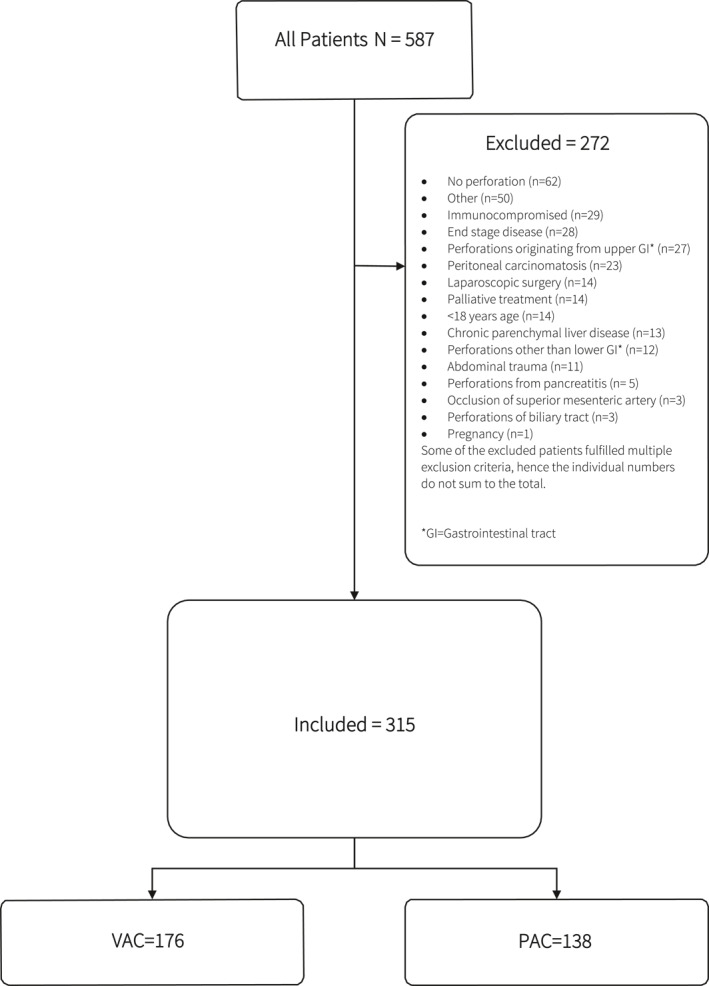
The flowchart of patient inclusion and exclusion.

Of the 587 registered patients, 315 met the inclusion criteria: 139 (44%) underwent PAC and 176 (56%) underwent VAC at the index operation (Table 1, Figure 1). The mean age was 66 ± 13 years in the PAC group and 63 ± 15 years in the VAC group. The VAC group exhibited significantly higher BMI, ASA, SOFA, and MPI scores at the index operation. However, no significant differences were observed between the groups regarding sex, age, Charlson comorbidity index, steroid use, or WHO performance status (Table 1). The median time from symptom onset to the operation was 24 h in the PAC group and 16 h in the VAC group [median (IQR): 27 (21–32) versus 22 (16–28), respectively; *p* = 0.527].

**TABLE 1:** Demographic data for the two groups primary abdominal closure (PAC) and vacuum‐assisted abdominal closure (VAC).

Demographic data	PAC *N* = 139	VAC *N* = 176	*p*‐value
Age in years	68 (59, 75)	67 (53, 74)	0.102
Female	69 (50%)	87 (49%)	1.000
Male	70 (50%)	89 (51%)	
Hours from debut of symptoms to operations	24 (9, 36)	16 (8, 36)	0.527
BMI^a^	24.7 (21.5, 28.1)	26.9 (23.4, 31.0)	< 0.001
Not reported	4	4	
ASA			
1	14 (10%)	5 (3%)	0.026
2	44 (32%)	44 (25%)	
3	63 (45%)	88 (50%)	
4	15 (11%)	30 (17%)	
5	1 (1%)	5 (3%)	
6	0 (0%)	0 (0%)	
Not reported	2 (1%)	4 (2%)	
Performance status			
0	52 (37%)	79 (45%)	0.319
1	50 (36%)	55 (31%)	
2	22 (16%)	17 (10%)	
3	4 (3%)	8 (5%)	
Not reported	11 (8%)	17 (10%)	
Steroid use			
No	128 (92%)	169 (96%)	0.169
Yes	10 (7%)	7 (4%)	
Not reported	1 (1%)	0 (0%)	
Previous abdominal operations			
No	40 (29%)	48 (27%)	0.605
Yes	98 (71%)	128 (72%)	
Not reported	1 (1%)	0 (0%)	
Charlson comorbidity index^a^	3 (2, 5)	3 (1.5, 5)	0.112
Charlson comorbidity index not reported	1	0	
Mannheim peritonitis index^a^	22 (16, 28)	27 (21, 32)	< 0.001
Mannheim peritonitis index not reported	1	0	
SOFA^a^	3 (1,78)	5 (2, 8)	0.002
SOFA not reported	41	19	

^a^ Figures are in median and interquartile range (brackets).

### 3.2 Surgical and Pathological Characteristics

The frequency of previous abdominal surgeries was similar between the groups (Table 2). One‐quadrant peritonitis was more frequently observed in the PAC group than in the VAC group (49% vs. 22%, respectively; *p* < 0.001), whereas four‐quadrant peritonitis was more common in the VAC group than in the PAC group (44% vs. 22%, respectively; *p* < 0.001). No significant differences were found in the anatomical location of the perforation. Bowel resection, anastomosis, placement of drainage, and stoma formation were significantly more common in the PAC group. In contrast, damage control and perforation suturing were significantly more frequent in the VAC group (Table 2).

**TABLE 2:** Perioperative findings and procedures.

Perioperative findings	PAC *N* = 139	VAC *N* = 176	*p*‐value
Contamination			
1 quadrant	68 (49%)	39 (22%)	< 0.001
2 quadrants	36 (26%)	52 (30%)	0.528
3 quadrants	1 (1%)	5 (3%)	0.234
4 quadrants	31 (22%)	77 (44%)	< 0.001
Not reported	3 (2%)	3 (2%)	1.000
Anatomical placement^a^			
Jejunum/ileum	55 (40%)	84 (48%)	0.171
Cecum	16 (12%)	18 (10%)	0.719
Ascending colon	7 (5%)	5 (3%)	0.380
Transvers colon	13 (9%)	18 (10%)	0.851
Descending colon	10 (7%)	12 (7%)	1.000
Sigmoid colon	33 (24%)	42 (24%)	1.000
Rectum	7 (5%)	7 (4%)	0.785
Not reported	2 (1%)	2 (1%)	1.000
Pathology^a^			
Perforation	64 (46%)	94 (53%)	0.213
Anastomotic leakage	40 (29%)	53 (30%)	0.805
Inflammation	19 (14%)	20 (11%)	0.606
Ischemia	9 (6%)	18 (10%)	0.311
Malign tumor	12 (9%)	7 (4%)	0.098
Other	1 (1%)	1 (1%)	1.000
Procedure^a^			
Bowel resection	123 (88%)	127 (72%)	< 0.001
Anastomosis	36 (26%)	22 (13%)	0.003
Placement of drainage	46 (33%)	13 (7%)	< 0.001
Stoma	95 (68%)	101 (57%)	0.048
Bowels left in discontinuity	4 (3%)	23 (13%)	0.001
Controlled fistulation	3 (2%)	1 (1%)	0.325
Other	2 (1%)	10 (6%)	0.073
Suture of perforation	11 (8%)	32 (18%)	0.008
Negative pressure			
< 125 mmHg		36 (20%)	
125 mmHg		138 (78%)	
> 125 mmHg		1 (1%)	
Not reported		1 (1%)	
Suture and type			
Continuous		47 (27%)	
Interrupted		114 (65%)	
Other		7 (4%)	
Monofilament absorbable		74 (42%)	
Monofilament nonabsorbable		86 (49%)	
Multifilament		3 (2%)	
Not reported		13 (7%)	
Malignancy			
No	121 (87%)	160 (91%)	0.308
Yes	17 (12%)	16 (9%)	
No reported	1 (1%)	0	

^a^ A patient can contribute to multiple of these categories.

### 3.3 Mortality Rate, CD Grade, and CCI Score

**FIGURE 2:**
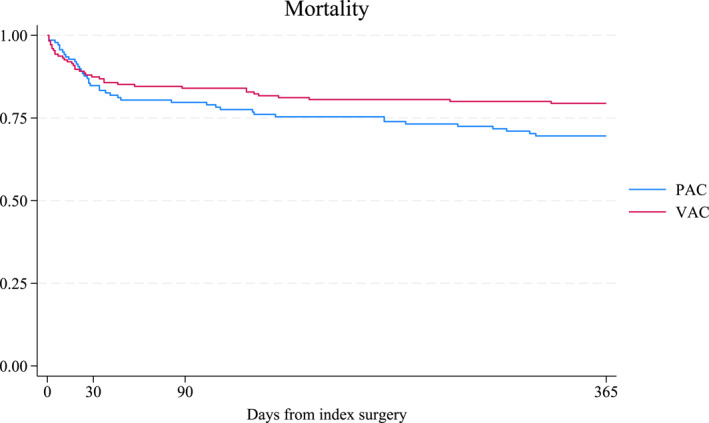
Kaplan–Meier survival curves showing mortality over time for patients with secondary peritonitis treated with primary closure (PAC) and vacuum‐assisted closure (VAC). The *x*‐axis represents days from the index surgery (0–365 days) and the *y*‐axis represents the cumulative survival probability (0.00–1.00).

There were no significant differences in postoperative mortality between the VAC and PAC groups at 30 days (13% vs. 16% and *p* = 0.519), 90 days (16% vs. 21% and *p* = 0.380), and 1 year (21% vs. 31% and *p* = 0.051) (Table 3.). The Kaplan–Meier curve for 1‐year mortality, shown in Figure 2, illustrates the cumulative survival trends between the groups. In‐hospital mortality did not significantly differ between the VAC and PAC groups (15% vs. 22% and *p* = 0.143; adjusted *p* = 0.292). In the unadjusted analysis, the hazard ratio for mortality associated with VAC was 0.66 (95% confidence interval: 0.42–1.03 and *p* = 0.067). After adjusting for confounding factors, the adjusted hazard ratio was 0.77 (95% confidence interval: 0.53–1.12 and *p* = 0.178).

**TABLE 3:** Postoperative outcomes.

Postoperative outcomes	PAC *N* = 139	VAC *N* = 176	*p*‐value	Adjusted *p*‐value
Mortality				
In‐hospital	31 (22%)	27 (15%)	0.143	0.2921
30 days	22 (16%)	23 (13%)	0.519	0.856
90 days	29 (21%)	29 (16%)	0.380	0.569
1‐year	43 (31%)	37 (21%)	0.051	0.116
CCI surgical^a^				
30 days	24.16 (22.27)	23.29 (21.45)	0.713	0.002
90 days	26.91 (22.49)	26.42 (24.19)	0.856	0.048
1‐year	30.85 (22.39)	30.55 (24.58)	0.909	0.009
CCI medical^a^				
30 days	45.76 (38.68)	52.52 (35.41)	0.050	0.247
90 days	47.14 (38.84)	55.42 (35.40)	0.046	0.357
1‐year	53.64 (39.78)	58.20 (34.61)	0.291	0.159
CCI total^a^				
30 days	55.56 (35.29)	61.62 (32.59)	0.101	0.065
90 days	57.92 (34.17)	64.04 (32.21)	0.099	0.209
1‐year	64.32 (33.90)	68.33 (29.78)	0.275	0.100
Fistula				
30 days	7 (6%)	10 (7%)	0.804	
90 days	12 (10%)	21 (14%)	0.353	
1‐year	14 (11%)	26 (17%)	0.174	
Wound abscess				
30 days	20 (16%)	26 (17%)	0.872	
90 days	21 (17%)	34 (22%)	0.291	
1‐year	24 (19%)	37 (24%)	0.313	
Secondary closure after VAC		165 (94%)		
Not reported		11 (6%)		
Secondary closure after PAC		139 (100%)		
Hernia at follow‐up				
Yes	38 (39%)	44 (34%)	0.577	
No	60 (61%)	84 (66%)		
Not reported	41	48		
Time to hernia (years)^a^	1.2 (0.85)	1.2 (0.79)	0.876	
Healthcare utility^a^				
Hospital stay (days)	26.3 (20.1)	28.8 (20.5)	0.178	
Intensive care unit stay (days)	6.7 (12.1)	9.0 (12.1)	< 0.001	
Resource use (US $)	146,019 (116,128)	164,906 (118,273)	0.079	
Reoperations excl. VAC changes	0.37 (0.66)	0.24 (1.12)	< 0.001	
CT‐scanning	1.2 (1.4)	1.4 (1.5)	0.267	
Percutaneous ultrasound guided drainage	0.23 (0.67)	0.31 (0.69)	0.159	

Abbreviation: CCI = comprehensive complication index.

^a^ Figures are mean and standard deviation (brackets).

Postoperative complications were assessed using both the CD classification and the CCI. According to the CD classification, patients in the PAC group had a significantly higher incidence of severe surgical complications (CD grade ≥ 3) (Table 4) and the VAC group exhibited significantly higher rates of severe medical complications (CD grade ≥ 3). Fascial dehiscence requiring treatment occurred more frequently in the PAC group than in the VAC group (22% vs. 9% and *p* = 0.003). Similarly, ileus requiring surgery was significantly more common in the PAC group compared to the VAC group (15% vs. 3% and *p* < 0.001). Among patients who underwent an anastomosis, the rate of grade ≥ 3b leakage was comparable between the two groups: 7 (19%) cases in the PAC group and 4 (18%) cases in the VAC group. By 90 days, no additional leakages were recorded. Within 1 year, only one additional case was documented in the VAC group.

**TABLE 4:** Postoperative complications within 1 year.

PAC										VAC									
Clavien–Dindo (grade)	0	1	2	3a	3b	4a	4b	5	≥ 3	0	1	2	3a	3b	4a	4b	5	≥ 3	*p* (for ≥ 3)
Bleeding	106	3	5	4	7	1	0	0	12 (10%)	132	3	7	2	6	1	4	1	14 (9%)	0.874
Fascial dehiscence	101	1	0	0	27	1	0	0	28 (22%)	139	0	0	0	14	0	0	0	14 (9%)	0.003
Ileus	108	0	0	1	17	1	0	0	19 (15%)	144	3	3	0	5	0	0	0	5 (3%)	< 0.001
Wound abscess	103	9	3	0	11	0	1	0	12 (9%)	117	20	2	2	13	0	0	0	15 (10%)	0.934
Stoma complication	104	6	3	2	13	0	0	0	15 (12%)	134	3	3	0	15	1	0	0	16 (10%)	0.694
Anastomotic leakage	114	0	1	1	8	3	0	0	12 (9%)	133	0	3	0	15	1	1	2	19 (12%)	0.453
Fistula	110	5	0	0	9	0	0	0	9 (7%)	125	5	3	4	14	0	0	0	18 (12%)	0.196
Others surgical	88	8	1	9	22	1	3	0	35 (27%)	112	6	3	6	34	2	1	2	45 (27%)	0.909
Stroke	122	1	2	1	0	0	0	3	4 (3%)	151	0	3	0	0	2	0	0	2 (1%)	0.287
AMI	128	0	0	1	0	0	0	0	1 (1%)	156	0	1	0	0	0	0	0	0 (0%)	0.269
Aspiration	116	0	5	0	0	3	6	0	9 (7%)	142	0	3	0	1	0	9	1	12 (8%)	0.816
Pneumonia	104	1	22	1	0	2	1	0	4 (3%)	117	2	30	3	0	6	0	1	10 (6%)	0.201
Cardiac failure	73	8	11	0	0	3	24	12	39 (30%)	70	6	13	1	0	19	39	20	79 (47%)	0.002
Pulmonary embolism	127	0	2	0	0	0	0	1	1 (1%)	152	0	3	0	0	0	1	1	2 (1%)	0.676
Respiratory failure	62	1	13	2	0	14	29	12	57 (43%)	60	0	26	4	1	19	48	9	81 (49%)	0.330
Kidney failure	78	20	4	0	0	8	22	1	31 (23%)	90	22	2	2	0	9	36	1	48 (30%)	0.222
Sepsis	64	1	24	0	1	9	28	7	45 (34%)	61	1	27	0	0	23	53	6	82 (48%)	0.012
Deep vein thrombosis	127	0	2	0	0	0	0	0	0 (0%)	150	0	6	0	0	0	0	0	0 (0%)	1.000
Arterial embolism	127	0	1	1	0	0	0	0	1 (1%)	156	0	1	0	0	0	0	0	0 (0%)	0.271
Others medical	39	16	64	5	2	2	0	7	16 (12%)	51	13	81	11	3	1	0	1	16 (10%)	0.597
Overall Clavien–Dindo ≥ 3									111 (80%)									146 (83%)	0.493
Overall surgical Clavien–Dindo ≥ 3									87 (64%)									89 (52%)	0.041
Overall medical Clavien–Dindo ≥ 3									72 (53%)									113 (65%)	0.028

For surgical complications, the adjusted CCI scores were significantly higher in the PAC group at all time points: 30 days (adjusted *p* = 0.002), 90 days (adjusted *p* = 0.048), and 1 year (adjusted *p* = 0.009). The unadjusted scores showed no significant differences between the groups at any time point.

For medical complications, the adjusted CCI scores did not reveal significant differences between the groups at any time point. However, the unadjusted scores showed a significantly higher medical CCI in the VAC group at 90 days (*p* = 0.046), with no significant difference at 30 days or 1 year.

For total CCI (surgical and medical combined), the adjusted and unadjusted scores showed no significant difference at any of the time points.

### 3.4 Intra‐Abdominal Abscess Requiring Percutaneous Drainage, Enteroatmospheric Fistula (EAF), and Wound Abscess

Twenty (15%) patients in the PAC group and 37 (21%) patients in the VAC group required percutaneous ultrasound‐guided drainage (PUD) for intra‐abdominal abscesses (*p* = 0.060). The mean number of PUDs per patient was similar in the PAC and VAC groups (0.23 ± 0.67 vs. 0.31 ± 0.69, respectively; *p* = 0.159) (Table 3). The cumulative incidence rates of EAF were similar between the PAC and VAC groups at all time points: 30 days (6% vs. 7%), 90 days (10% vs. 14%), and 1 year (11% vs. 17%) (Table 3). Wound abscess rates following abdominal closure were also comparable between the two groups across all time points: 30 days (16% vs. 17%), 90 days (17% vs. 22%), and 1 year (19% vs. 24%) (Table 3).

### 3.5 VAC Changes and Abdominal Closure After VAC

The median number of scheduled VAC changes was 2 (IQR: 1–3). Negative pressure of 125 mmHg was applied in most cases (78%), with 20% receiving < 125 mmHg and 1% receiving > 125 mmHg (Table 2). Abdominal pressure data were unavailable for 1% of the patients. Secondary fascial closure was achieved in 94% of patients treated with VAC, whereas data were not reported for the remaining 6%. In the PAC group, 100% was closed. Fascial closure was achieved using interrupted sutures in 65% of patients, continuous sutures in 27%, and other methods in 4% (Table 2). Absorbable monofilament were used in 42% and nonabsorbable monofilament sutures were used in 48% of patients, with multifilament sutures used in 2%. Abdominal closure following VAC treatment occurred after a median of 4 days (IQR: 3–7.5 days).

### 3.6 Hospital and ICU Stays, Reoperations, and Resource Use

The mean hospital stay was 26.3 ± 20.1 days in the PAC group and 28.8 ± 20.5 days in the VAC group (*p* = 0.178) (Table 3). The mean ICU stay was 6.7 ± 12.1 days in the PAC group and 9.0 ± 12.1 days in the VAC group (*p* ≤ 0.001). Unscheduled reoperations were required in 64 (48%) patients in the PAC group and 48 (28%) patients in the VAC group (*p* < 0.001). The mean number of unscheduled reoperations was higher in the PAC group than in the VAC group (0.37 ± 0.66 vs. 0.24 ± 1.12, respectively; *p* ≤ 0.001). In the PAC group, 35 (25%) patients required later conversion to VAC therapy.

There was considerable variability in the use of VAC and PAC across centers. VAC usage ranged from 26.9% to 87.5%, whereas PAC usage varied from 12.5%% to 73%, indicating a preference for VAC in multiple centers. In secondary centers, 47 of 55 (85%) patients received VAC compared with 131 of 260 (50%) patients at the tertiary center (*p* < 0.001). However, because of the limited number of cases in most centers outside the tertiary center, statistical analysis of center‐specific variability was not feasible.

Total hospital costs were similar in the PAC and VAC groups (mean of 146,019 ± 116,128 vs. 164,906 ± 118,723 United States dollars, respectively) (*p* = 0.079).

### 3.7 CT Scans

The mean number of postoperative CT scans was 1.2 ± 1.4 in the PAC group and 1.4 ± 1.5 in the VAC group (*p* = 0.267) (Table 3).

### 3.8 Ventral Hernia

The incidence of ventral hernia at 1 year was 39% in the PAC group and 34% in the VAC group (*p* = 0.577) (Table 3).

## 4 Discussion

The mortality rates in our study did not significantly differ between the VAC and PAC groups at 30 days, 90 days, and 1 year. However, a trend toward lower 1‐year mortality in the VAC group was observed (21% vs. 31% and *p* = 0.051).

Patients in the VAC group exhibited significantly higher baseline disease severity, as indicated by elevated ASA, SOFA, and MPI scores as well as the presence of four‐quadrant contamination. This heightened severity may account for the increased incidence of severe medical complications, such as sepsis and cardiac failure, observed in the VAC group. Consequently, the VAC group experienced longer ICU stays. Additionally, unscheduled reoperations were significantly more frequent in the PAC group, with 25% of PAC‐treated patients requiring conversion to VAC therapy during their treatment course, emphasizing the potential advantages of VAC in managing complex secondary peritonitis cases.

Other studies have investigated mortality rates in patients with secondary peritonitis treated with either VAC or PAC. In our study, the 1‐year mortality rate in the PAC group was 31%, similar to the rate of 29% reported in a randomized trial comparing on‐demand versus planned relaparotomy strategies in patients with secondary peritonitis (15). A systematic review and meta‐analysis focusing on nontrauma patients treated with various VAC techniques reported slightly higher mortality rates, ranging from 21.5% to 30.0%, depending on the specific method used. These findings are consistent with the 21% 1‐year mortality observed in the VAC group within this study [32]. In a separate retrospective analysis, involving 438 patients treated with VAC for abdominal emergencies—67% of whom had secondary peritonitis—the mortality rates were 14% at 30 days, 21% at 90 days, and 31% at 1 year [40]. In comparison, we observed lower rates of 13%, 16%, and 21% at these respective time points.

Additional comparative studies have reported varying results. In one retrospective study, the in‐hospital mortality rate was 22.8% for VAC‐treated patients and 38.6% for PAC‐treated patients, though only 58.2% of these patients had secondary peritonitis and were treated for severe nontraumatic sepsis or septic shock following urgent laparotomy [38]. Another retrospective cohort study involving 203 VAC‐treated and 331 PAC‐treated patients with secondary peritonitis revealed an inpatient mortality rate of 22.5% in the VAC group and 11.7% in the PAC group, findings that contrast with our results [45].

This discrepancy may reflect differences in patient characteristics because the VAC group in that study included older patients, patients transferred from other facilities due to the complexity of their condition, and patients more frequently undergoing night‐time surgeries. Additionally, VAC‐treated patients in that cohort required greater ionotropic support and blood transfusions. In our study, significant differences in disease severity were also observed between the VAC and PAC groups.

Although no significant differences in survival were observed between the VAC and PAC groups. One notable factor that needs to be considered is the fewer anastomoses performed in the VAC group compared with the PAC group (13% vs. 26% patients, respectively). Avoiding anastomoses is a well‐recognized strategy to reduce the risk of severe complications, such as anastomotic leakage, which can exacerbate sepsis and precipitate multiorgan dysfunction. These differences in operative decision‐making represent an important confounding factor that must be considered when interpreting our results.

The incidence of intra‐abdominal abscesses and the need for percutaneous ultrasound‐guided drainage were similar between the VAC and PAC groups despite the higher frequency of four‐quadrant peritonitis and greater SOFA and MPI in the VAC group. This might also indicate that VAC therapy is associated with improved management of peritoneal contamination. However, this finding contrasts with the results reported by Kao et al. [45], who observed a high complication rate of 71.2% among patients managed with the VAC approach. The adjusted analysis of postoperative complications using the CCI revealed significant differences between the groups at 30 days, 90 days, and 1 year. Surgical complications classified as CD grade ≥ 3 were significantly lower in the VAC group, with an incidence of 52% compared to 64% in the PAC group. These findings align with prior studies, such as Bensignor et al. [35], who reported a 47% rate of CD grade > 2 complications, and Van Ruler et al. [16], whose randomized controlled trial revealed a 40% incidence of peritonitis‐related complications after 1 year. Furthermore, the PAC group demonstrated significantly higher rates of fascial dehiscence and ileus requiring surgical intervention compared to the VAC group. These findings highlight VAC therapy's effects on postoperative outcomes, potentially lowering certain surgical complications in more severely ill patients.

The 1‐year incidence rates of EAF were comparable between the groups (11% in the PAC group and 17% in the VAC group). These rates are similar to the 12% rate reported among 332 patients with peritonitis treated with VAC [66]. It has been suggested that the risk of EAF may be more closely associated with the underlying condition than with the specific closure technique employed [66]. However, the incidence of EAF in patients with peritonitis treated with PAC is not well documented. One larger retrospective study reported an incidence of 9.6% [46]. For patients treated with VAC, the reported incidence ranges widely from 1% to 37% [34, 37, 39, 40, 41, 44, 46, 48, 50, 51, 53, 54, 58, 59, 60, 61].

The observed rise in the number of EAFs over the first year likely reflects multiple factors, including the closure of temporary stomas or anastomoses, which are recognized risk factors for fistula formation. Additionally, the cumulative nature of the 1‐year incidence data includes all events occurring throughout the year contributing to the appearance of a gradual increase.

Secondary fascial closure was achieved in 94% of patients treated with VAC. A systematic review revealed rates ranging from 51.5% to 73.1% depending on the VAC technique used [32]. In our analysis, the incidence of incisional hernias at 1 year was 39% in the PAC group and 34% in the VAC group, with no significant difference between the two. Data on the incidence of hernias following PAC after secondary peritonitis are notably sparse, with one study reporting a rate of 20.8% [46]. The reported rates after VAC treatment vary widely, ranging from 3.4% to 48.1% [39, 40, 52, 54]. This variation highlights the need for more comprehensive reporting and analysis to better understand the factors influencing hernia development. It also suggests the value of a longer follow‐up period of at least 3 years.

This study has several limitations. As a retrospective chart review, it inherently lacks randomization and is susceptible to selection bias. This nonrandomized approach introduces potential confounding variables that could influence mortality and morbidity outcomes. Despite employing robust data collection methods, retrospective studies carry the risk of incomplete or inaccurate data. To mitigate this risk, records were reviewed twice by two independent authors to minimize data entry errors.

Our findings may be influenced by variability in VAC and PAC usage across centers, as the choice of treatment was based on surgeon discretion. This variation reflects differences in local practices and potential surgeon preferences. Additionally, the VAC group had a higher frequency of four‐quadrant contamination, ASA, SOFA and higher MPI scores, indicating that VAC was more frequently used in patients with more severe peritonitis, consistent with current guideline recommendations.

An important limitation of this study is the regional variation in VAC use, which may introduce bias and limit the generalizability of our findings to other settings. Another limitation is the inability to determine the exact causes of mortality during the 30‐day, 90‐day, and 1‐year follow‐up periods. Although it is likely that many deaths were attributable to sepsis‐related organ failure and multiorgan dysfunction syndrome, distinguishing whether mortality resulted from complications of the index operation, progression of the underlying disease, or comorbidities remains challenging.

In this study, we categorized the etiology of secondary peritonitis based on primary pathological causes rather than specific diagnoses. This approach was chosen to better reflect the clinical relevance of the underlying pathology and to facilitate meaningful comparisons between groups. Although each patient had a specific diagnosis, recording an individual diagnosis for each patient would have introduced significant variability, making it difficult to analyze or compare data across groups. Our data reflect the main categories of pathology (e.g., perforation, inflammation, ischemia, and malignancy) and include whether each case was malignant or benign. However, this structured approach limits our ability to provide detailed ICD‐10‐based diagnoses for each patient, which we acknowledge as a limitation.

Our dataset also lacks detailed information on delayed anastomosis within the VAC group because our primary aim was to compare major outcomes rather than specific procedural details. Only a small percentage (13%) of cases involved damage control surgery, reflecting a mixed population rather than a focused study on damage control surgery or VAC. Furthermore, our dataset did not include information on the timing or circumstances of care withdrawal, which may influence mortality outcomes and the decision between PAC and VAC. This limitation should be considered when interpreting our findings.

The incomplete documentation of APACHE II scores is another limitation. Although such scores could provide valuable insights into disease severity, retrospectively reconstructing them from incomplete patient data were deemed unreliable and prone to bias. We chose to exclude these scores from our analysis to maintain the integrity of our findings.

Further research, particularly multi‐institutional trials with standardized protocols, is crucial for refining these management strategies and improving patient outcomes. Ongoing randomized controlled trials are expected to provide valuable data to support evidence‐based practices in this field [62, 63].

## 5 Conclusion

This retrospective comparative study demonstrated no significant differences in mortality rates between VAC and PAC in the treatment of secondary peritonitis. VAC was associated with fewer surgical complications and a lower incidence of surgical CD grade ≥ 3 complications. Well‐designed randomized studies are needed to validate these findings and further explore the potential benefits of VAC therapy.

## Author Contributions

**Pooya Rajabaleyan:** conceptualization, data curation, formal analysis, methodology, validation, writing–original draft, writing–review and editing. **Ask Vang:** data curation, formal analysis, writing–review and editing. **Sören Möller:** conceptualization, formal analysis, methodology, visualization, writing–review and editing. **Sardar Khalaf:** data curation, formal analysis, writing–review and editing. **Anna Gosvig Ladegaard:** data curation, formal analysis, writing–review and editing. **Niels Qvist:** conceptualization, formal analysis, methodology, writing–review and editing. **Mark Bremholm Ellebæk:** conceptualization, formal analysis, methodology, validation, writing–review and editing.

## Ethics Statement

This study was approved by the Regional Data Protection Agency (project ID: 21/21873) and the Regional Committees on Health Research Ethics for Southern Denmark (project ID: 21/20419) and was performed in accordance with the Declaration of Helsinki.

## Conflicts of Interest

The primary investigator and collaborators declare that they have no financial interest in the trial.

## Supporting information

Supporting Information S1

## Data Availability

Data can be shared anonymously upon reasonable request, provided a data processor agreement is obtained.
