# Supplementary material for: Vacuum‐Assisted Closure Significantly Reduces Surgical Postoperative Complications Compared With Primary Abdominal Closure in Patients With Secondary Peritonitis: A Comparative Retrospective Study
Source: World J Surg. 2025 Jan 10;49(2):387–400. doi: 10.1002/wjs.12472 (PMC11798683; doi:10.1002/wjs.12472)
Supplement: Supplementary file 1 — Supporting Information S1 [file WJS-49-387-s001.docx]

# Appendix 1: Table of ICD-10 diagnosis codes

| Condition | Diagnosis Codes | Procedure Codes |
| --- | --- | --- |
| Meckel’s (*) | T00713 DQ430 | KJFB KJFB96 KJH00 BNPA92 KJAH30 KJFH20 KJFH KJAH33 KJFH10 KJ6B10 KJFH00 |
| Small bowel volvulus (*) | DK562C | KJFB KJFB96 KJH00 BNPA92 KJAH30 KJFH20 KJFH KJAH33 KJFH10 KJ6B10 KJFH00 |
| Anastomotic leakage | DT813A DT813A1 DT813A2 DT813G | KJWF KJWF00 KJFB KJFB20 KJFB96 KJH00 KJFB46 KJFB30 KJFB43 KJFB56 KJFB30B KJFH96 KJFH01 BNPA92 KJAH30 KJFH20 KJFH KJAH33 KJFH10 KJ6B10 KJFH00 |
| Perforation of small intestine, colon, or rectum | DK631 DK631A DK628H | KJFB KJFB96 KJFB20 KJFB96 KJH00 KJFB46 KJFB30 KJFB43 KJFB56 KJFB30B KJFH96 KJFH01 KJH00 KJGB10 KJGB40 KJGB00 KJGB30 KJGB31 BNPA92 KJAH30 KJFH20 KJFH KJAH33 KJFH10 KJ6B10 KJFH00 |
| Diverticulitis of small or large intestine with perforation | DK570 DK570B DK570C DK572B DK572C DK574 DK574A | KJFB KJFB20 KJFB96 KJH00 KJFB46 KJFB30 KJFB43 KJFB56 KJFB30B KJFH96 KJFH01 BNPA92 KJAH30 KJFH20 KJFH KJAH33 KJFH10 KJ6B10 KJFH00 |
| Volvulus (*) | DK562 DK652B | KJFB KJFB20 KJFB96 KJH00 KJFB46 KJFB30 KJFB43 KJFB56 KJFB30B KJFH96 KJFH01 BNPA92 KJAH30 KJFH20 KJFH KJAH33 KJFH10 KJ6B10 KJFH00 |
| Ileus (*) | DK567 | KJFB KJFB96 KJWF KJWF00 KJFB KJFB20 KJFB96 KJH00 KJFB46 KJFB30 KJFB43 KJFB56 KJFB30B KJFH96 KJFH01 BNPA92 KJAH30 KJFH20 KJFH KJAH33 KJFH10 KJ6B10 KJFH00 |
| Hernia with ileus (*) | DK420 DK430 DK400 DK403 DK403A DK403B DK433 DK450 DK450C DK450F DK460 | KJFB KJFB96 KJWF KJWF00 KJFB KJFB20 KJFB96 KJH00 KJFB46 KJFB30 KJFB43 KJFB56 KJFB30B KJFH96 KJFH01 BNPA92 KJAH30 KJFH20 KJFH KJAH33 KJFH10 KJ6B10 KJFH00 |
| Ischemic colitis (*) | DK550A | KJFB20 KJFB96 KJH00 KJFB46 KJFB30 KJFB43 KJFB56 KJFB30B KJFH96 KJFH01 KJH00 BNPA92 KJAH30 KJFH20 KJFH KJAH33 KJFH10 KJ6B10 KJFH00 |
| Perforation after screening colonoscopy (*) | DT812G1 | KJFB20 KJFB96 KJH00 KJFB46 KJFB30 KJFB43 KJFB56 KJFB30B KJFH96 KJFH01 KJH00 KJGB10 KJGB40 KJGB00 KJGB30 KJGB31 BNPA92 KJAH30 KJFH20 KJFH KJAH33 KJFH10 KJ6B10 KJFH00 |
| Peritonitis | DK659 DK650 DK650M DK650N DK658 DK650P | KJFB KJFB96 KJFB20 KJFB96 KJH00 KJFB46 KJFB30 KJFB43 KJFB56 KJFB30B KJFH96 KJFH01 KJH00 KJGB10 KJGB40 KJGB00 KJGB30 KJGB31 BNPA92 KJAH30 KJFH20 KJFH KJAH33 KJFH10 KJ6B10 KJFH00 |

(*) Must be registered with one of these: DK650 DK650P DK659 DK650M DK650N
